# *QuickStats*: Age-Adjusted Motor Vehicle Traffic Death Rates,*^,^^†^ by Urban-Rural Status^§^ and Sex — National Vital Statistics System, United States, 2017

**DOI:** 10.15585/mmwr.mm6825a4

**Published:** 2019-06-28

**Figure:**
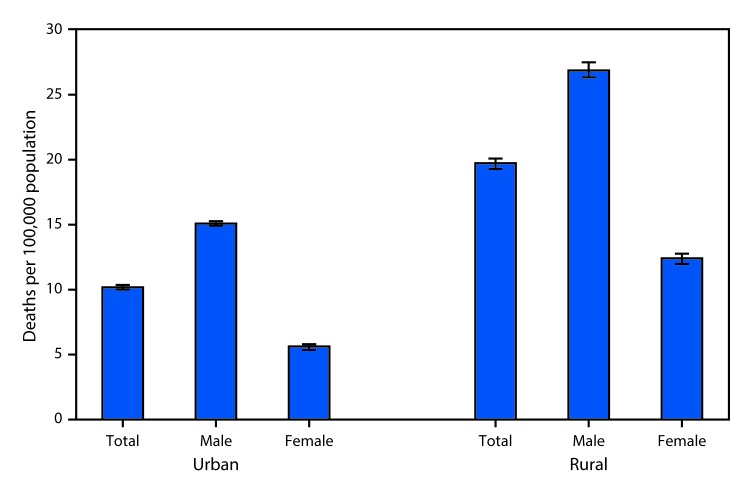
In 2017, the age-adjusted rate of motor vehicle traffic deaths was higher for residents of rural areas (19.7 per 100,000) than urban areas (10.2). Rates were higher in rural compared with urban areas for both female and male residents, and rates for males were higher than for females in both urban and rural areas. The death rates were 12.6 per 100,000 for female residents of rural areas, 5.6 for female residents of urban areas, 26.9 for male residents of rural areas, and 15.1 for male residents of urban areas.

**Sources:** National Center for Health Statistics, National Vital Statistics System, Mortality File. https://www.cdc.gov/nchs/nvss/deaths.htm. Ingram DD, Franco SJ. 2013 National Center for Health Statistics urban-rural classification scheme for counties. Vital Health Stat 2014;2(166). https://www.cdc.gov/nchs/data/series/sr_02/sr02_166.pdf.

For more information on this topic, CDC recommends the following link: https://www.cdc.gov/motorvehiclesafety/calculator/index.html.

